# Large Deformation and Energy Absorption Behaviour of Perforated Hollow Sphere Structures under Quasi-Static Compression

**DOI:** 10.3390/ma14133716

**Published:** 2021-07-02

**Authors:** Meiling Dai, Junping Liang, Cheng Cheng, Zhiwen Wu, Jiexun Lu, Jiyu Deng

**Affiliations:** 1 School of Civil and Transportation Engineering, Guangdong University of Technology, Guangzhou 510006, China; meiling-dai@163.com (M.D.); supclot@163.com (J.L.); 1216416395cchh@gmail.com (C.C.); 15088195692@163.com (Z.W.); a18819161330@163.com (J.L.); 2 School of Architecture and Urban Planning, Guangdong University of Technology, Guangzhou 510090, China

**Keywords:** cellular material, hollow sphere structure, compression test, numerical simulation, large deformation, energy absorption

## Abstract

Hollow sphere structures with perforations (PHSSs) in three different arrangements (simple cubic (SC), body-centred cubic (BCC), and face-centred cubic (FCC)) were fabricated through three-dimensional (3D) printing, and the mechanical behaviours of these PHSSs under quasi-static compression were investigated experimentally and numerically. The results indicated that under uniaxial compression, the PHSSs mainly undergo three stages, i.e., a linear elastic stage, a large deformation or plateau stage, and a densification stage. During the stage of large deformation, the SC and BCC PHSSs experience a preliminary compaction sub-stage after layer-by-layer buckling, while for the FCC PHSS, layer-by-layer collapse and compaction are the dominant deformation behaviours. A numerical simulation was employed to study the mechanical properties of PHSSs with different geometric parameters under quasi-static compression and to explore the effect of the wall thickness, hole diameter, and sphere arrangement on the first peak stress, plateau stress, and specific energy absorption (SEA) of the PHSSs. The results reveal that the geometric parameters have a significant impact on the large deformation behaviour and energy absorption capacity of PHSSs. The presented PHSS is also proven to be much lighter than traditional metallic hollow sphere structure (MHSS) and has higher specific strength and SEA.

## 1. Introduction

With abundant pores in the matrix material and an internal microstructure, cellular metal or metal foam has unique functional and structural properties. This material is ultralightweight and is characterised by good sound absorption, high heat resistance, energy absorption, and high specific stiffness and strength [1]. It has been used extensively in engineering fields, such as aerospace, military, and transportation engineering. A metallic hollow sphere structure (MHSS) is a high-quality cellular material that is usually manufactured by filling thin-walled hollow spheres into a polymer matrix, sintering the spheres by applying heat and pressure and bonding spheres using a liquid phase [2]. In addition to the advantages of conventional cellular metals, MHSSs are characterised by fewer defects and more uniform geometry, resulting in more consistent mechanical properties [3], and have drawn the attention of a large number of researchers.

Based on the regular atomic arrangement concept, Sanders and Gibson performed a numerical simulation analysis of the elasticity modulus and initial yield stress of MHSSs in different packing patterns, such as simple cubic (SC), body-centred cubic (BCC) and face-centred cubic (FCC) patterns, and found that MHSSs have better mechanical properties than cellular materials with random porous structures [4,5]. Gasser et al. used a similar method to determine the tensile elastic constant of a regularly stacked (FCC) brazed MHSS; they also analysed the effects of the thickness-sphere radius ratio and the weld radius-sphere radius ratio on the overall elastic modulus and established an empirical formula for the nominal elasticity modulus [6,7]. Fiedler and Aöchsner et al. studied the mechanical properties of composite MHSSs and partially bonded MHSSs in an SC arrangement as well as the mechanical properties and size effect of sintered MHSSs [8,9,10]. Shufrin et al. derived a three-dimensional (3D) continuum model to analyse the negative Poisson’s ratio (NPR) behaviour of hollow sphere structures and evaluated the effect of the arrangement of hollow spheres on the NPR behaviour through numerical simulations [11].

Many experimental and numerical studies have also been performed to reveal the large deformation process and quasi-static and dynamic responses of hollow sphere structures in detail. Gao et al. numerically studied the static and dynamic deformation behaviours and mechanical properties by using simplified geometric models of sintered MHSSs and proposed formulas for the elastic modulus, yield strength, and plateau stress of the FCC packing material under quasi-static compression [12]. Vesenjak et al. studied the dynamic mechanical behaviours of composite MHSSs and partially bonded MHSSs in different packing patterns under impact loading through numerical simulation [13]. Cunda et al. analysed the differences in the mechanical properties of MHSSs under static and impact loadings using the finite element method and evaluated the effect of the loading rate on the dynamic mechanical behaviours and energy absorption capacity [14]. Marcadon et al. studied the compressive mechanical properties and creep behaviours of MHSSs under quasi-static loading and the effect of geometric defects on these mechanical properties [15,16]. Most studies used representative volume element (RVE) analyses to avoid considerable computing time for the simulation; however, these models cannot reveal the deformation mechanism of a real material due to the localization of deformation at large strains.

With ping-pong balls as the experimental object, Yu et al. carried out a series of static and dynamic compression experiments on single-ball and multi-ball arrays, theoretically derived the compressive deformation pattern of a stacking of spheres, and verified the theories through various two-dimensional (2D) ping-pong ball stackings; some findings were beneficial to understanding the deformation mechanism of a real MHSS [17,18,19]. They also conducted experimental studies through quasi-static tests and dynamic tests to investigate the large deformation behaviour of sintered MHSSs, and a long plateau length (up to 67% of the nominal strain) and localisation of deformation were observed [20]. Luo et al. studied the crushing performance of thin-walled tubes filled with MHSs under axial compression; the results indicated that the MHSs significantly improved the axial bearing capability of thin-walled tubes [21]. Zeng et al. conducted experimental studies on the dynamic mechanical properties of MHSS with density gradients [22]. However, due to the limitation of the specimen, the influence of the arrangement of hollow spheres on the mechanical properties could not be examined experimentally.

Although some understanding of the static and dynamic mechanical properties of hollow sphere structures/materials has been gained through these studies, which were mostly conducted under ideal stacking and connection conditions by using the finite element method, the obtained theoretical formulas are not consistent with the experimental results in some cases. Although not impossible, it is at least difficult for traditional manufacturing technology to ensure that the stacking pattern of hollow spheres is kept in line as well as the connection mode between spheres; moreover, the mechanical properties of hollow sphere materials are extremely sensitive to the arrangement pattern of the spheres and the connection geometry between the spheres.

Additive manufacturing/3D printing, as an emerging manufacturing technology, can be used to directly produce more complex components regardless of the structure or shape, and this technique offers a new approach for fabricating hollow sphere materials. Recently, researchers have used stainless steel, photosensitive resin, polylactic acid (PLA) plastics, and nylon to fabricate different hollow sphere structures through additive manufacturing techniques, such as fusion selective laser melting (SLM) [23,24], stereolithography (SLA) [25], fused deposition modelling (FDM) [26], and multi-jet fusion (MJF) [27], and carried out a series of experiments to investigate the mechanical properties of these structures, most of which introduced perforations or open porosities in spherical walls to remove the excess material inside the structure during post-processing of 3D printing; however, these perforations in hollow spheres change the physical and mechanical properties of hollow sphere structures to some degree. Parameter studies have also been conducted by using finite element models (FEMs) to quantitatively analyse the effect of geometric parameters on the structural mechanical properties [25,27]. However, these studies focused on the elastic and initial yielding stages because of the dramatic increase in the computational time in the simulation of large plastic deformations.

Understanding the mechanism of large deformation or plastic behaviour of perforated hollow sphere structures (PHSSs) is important for designers to more fully utilise the energy absorption characteristics. In this work, multi-cell PHSSs in different arrangements were prepared by using the MJF 3D printing technology and the large deformation behaviour of PHSSs under quasi-static compression was then investigated experimentally and numerically. The experimental results verified the effectiveness of the associated FEMs. Furthermore, the effect of geometric parameters, such as wall thickness, hole diameter and packing pattern, on the structural deformation mode, first peak stress, plateau stress and specific energy absorption (SEA) was analysed by using the finite element method, which offers a reference for further lightweight engineering applications of 3D printed hollow sphere structures.

## 2. Materials and Methods

**Figure 1:**
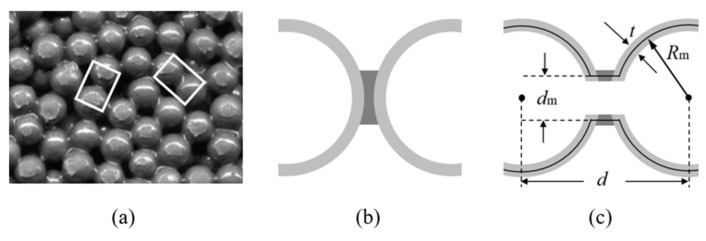
(**a**) Traditional MHSS; structure of two connected spheres (**b**) without and (**c**) with perforations.

**Figure 2:**
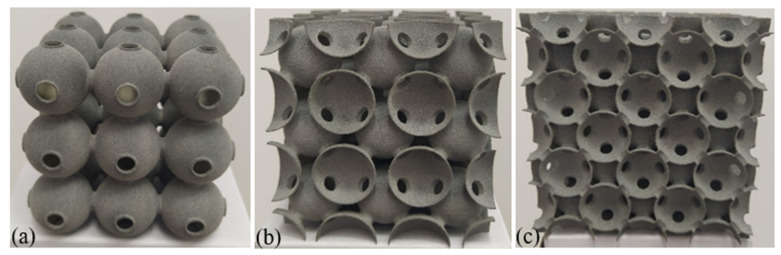
A 3D printed PHSS: (**a**) SC; (**b**) BCC; (**c**) FCC.

For the traditional soldered or adhesive bonded metallic hollow-sphere structure (Figure 1a), the connection part between two spheres adopts a certain geometry, as shown in Figure 1b. For the PHSSs, the connection cavity shown in Figure 1c was used to link two perforated spheres, and the wall thickness of the connection cavity was identical to that of a hollow sphere; this geometric model can be divided into appropriate shell elements to reduce the simulation time significantly [12]. The main geometric parameters include the hollow sphere radius *R_m_*, wall thickness *t*, opening or hole diameter *d_m_*, and distance *d* between adjacent hollow spheres. Since the perforations are located between spheres or in connections, for different sphere spatial arrangements, the numbers of openings in a single hollow sphere are different: there are 6, 8 and 12 openings corresponding to 6, 8 and 12 connections for one single hollow sphere in the SC, BCC and FCC packing structures, respectively. Figure 2a–c shows the 3D printed 3 × 3 × 3-cell PHSSs in different arrangements, where *R*_m_ = 9.75 mm, *d* = 20.5 mm, *d*_m_ = 5.5 mm, and *t* = 0.5 mm, and the relative density is 0.0654, 0.0833, and 0.0882, respectively. The relative density can be calculated by Equation (1) [25].

(1) $$\rho_{rel}=\frac{V_{So}}{V_{UC}}$$

where $V_{So}$ is the volume of the solid material and $V_{UC}$ is the volume of the unit cell or the entire specimen. $V_{So}$ and $V_{UC}$ can be obtained from the 3D design software Rhino.

In this paper, HP MJF 3D printing technology is used to produce the experimental samples, and HP nylon P12 powder is used as the base material. Under the standard printing process, the MJF 3D printing accuracy is 0.1 mm, and the density of the printed solid material is 1.01 g/cm^3^; this material can be simplified as an ideal elastoplastic material with a Young’s modulus of 1700 MPa and a yield stress of 48 MPa [27,28].

To ensure the reliability of the experimental results, three samples of each packing pattern were prepared and tested in this study. After printing, all the hollow sphere structures stood for 10 days in a dry environment at room temperature (25 °C) and were then subjected to a quasi-static compression experiment. The samples were placed between the upper and lower plates of a hydraulic universal testing machine (Changchun DDL100, China) with an accuracy of 0.5% in the experiment, and the downward loading rate of the upper plate was set to 10 mm/min.

**Figure 3:**
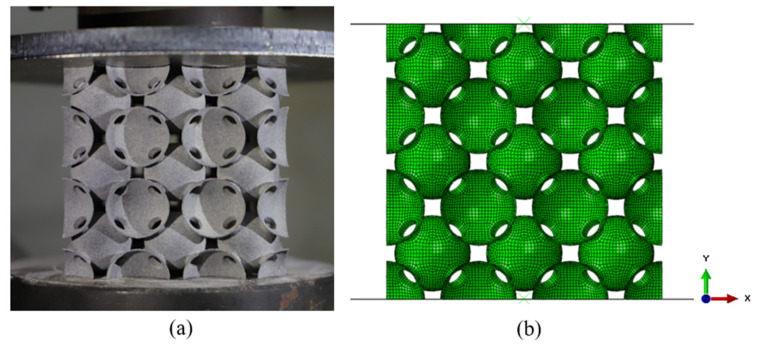
(**a**) Experimental model and (**b**) FEM of the BCC structure.

Numerical simulations were performed for the structures under the corresponding experimental conditions using ABAQUS 6.14 finite element software. The geometric models of PHSSs in different arrangements were simulated with automatically generated four-node shell element (S4R) meshes, which significantly reduced the modelling time. An average length of the elements 0.8 mm, approximately 4% of the sphere diameter, was used following a mesh convergence analysis. For the SC, BCC, and FCC PHSSs with 3 × 3 × 3 cells, the FEMs contained 53,352, 96,336, and 189,216 elements, respectively. The FEM of the BCC PHSS is shown in Figure 3 as an example. The bottom plate was subjected to fixed restraints, a vertical downward displacement load was arranged at the top plate, and the contact between the rigid plates and the structure was defined as surface-to-surface contact. The hard contact algorithm was used for normal behaviour, and a friction coefficient of 0.1 was considered for tangential behaviour. In consideration of the self-contact behaviour in the large deformation stage, general contact was used for all structures. 

The ABAQUS/Explicit method was then employed to simulate the mechanical behaviour of different structures under quasi-static compression, and a comparison was made between the numerical simulations and the experimental results. For ease of analysis, the nominal stress *σ* was defined as the plate pressure divided by the projected area of the structure on the plane, and the nominal strain *ε* was defined as the ratio of the loading displacement to the initial height of the structure. It is necessary to note that all the “stress” and “strain” mentioned in following sections indicate the nominal stress and nominal strain respectively, if not indicated otherwise.

A parameter study was conducted to assess the effect of wall thickness *t* and hole diameter *d_m_* on the compressive mechanical properties and energy absorption capacity of the structures. The relationships between the geometric parameters and the relative density are shown in Table 1. For all geometric models, the sphere radius *R_m_* was fixed to 9.75 mm, and the distance between adjacent hollow spheres was fixed to 20.5 mm. As *t* varied (*t* = 0.195~0.975 mm), *d_m_* was fixed to 5.5 mm. As *d_m_* varied (*d_m_* = 3.5~7.5 mm), *t* was fixed to 0.5 mm. As shown in Table 1, the *t* value has a significant effect on the relative density, while the *d_m_* value has a small effect on the density. In addition to the relative density, the thickness-to-radius ratio (*t/R_m_*) is also an important parameter of hollow spheres [12]. According to Table 1, when *d_m_* = 5.5 mm, there is an approximately linear relationship between *t/R_m_* and the relative density by using the linear fitting method: $\rho_{SC}\approx 1.275\cdot \left(t/R_{m}\right)$; $\rho_{BCC}\approx 1.622\cdot \left(t/R_{m}\right)$; and $\rho_{FCC}\approx 1.712\cdot \left(t/R_{m}\right)$. The fitting accuracy is above 98%.

## 3. Results and Discussion

### 3.1. Mechanical Behaviour of the PHSS

**Figure 4:**
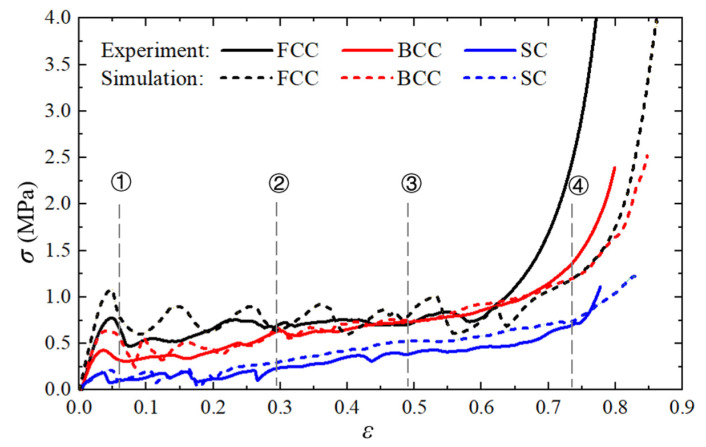
Nominal stress–strain curves of different structures.

**Figure 5:**
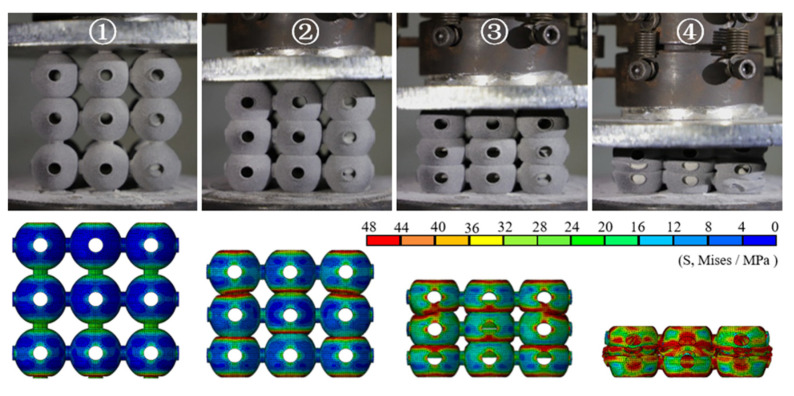
Deformed SC structures at different stages.

**Figure 6:**
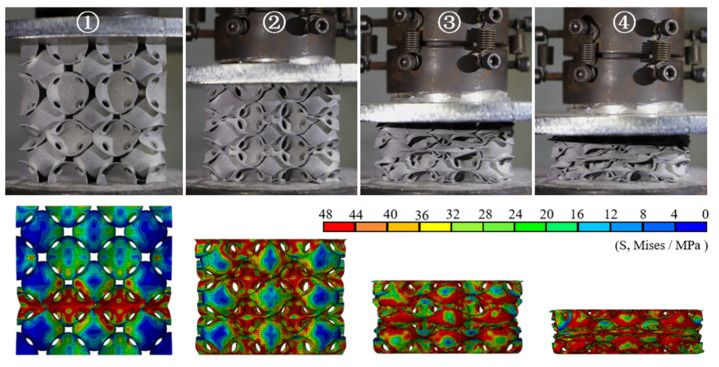
Deformed BCC structures at different stages.

**Figure 7:**
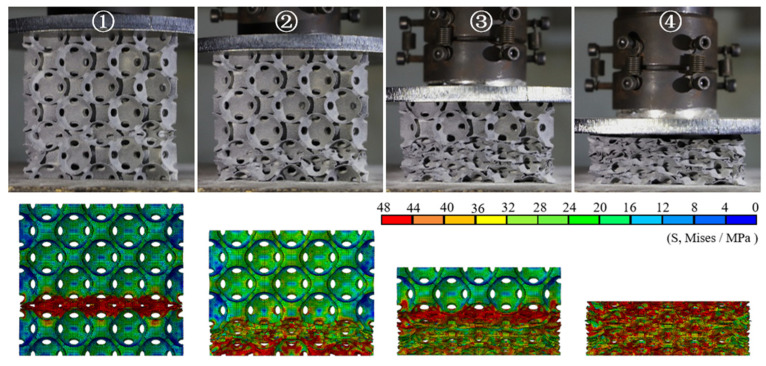
Deformed FCC structures at different stages.

**Figure 8:**
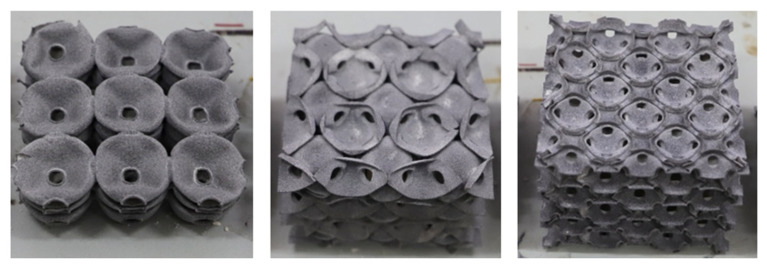
Compressed hollow sphere structures.

Figure 4 shows the nominal stress–strain curves of the different PHSSs, and Figure 5, Figure 6 and Figure 7 show the large deformation process of the SC, BCC, and FCC PHSSs, respectively. According to the experimental results and numerical simulations, similar to the compressive mechanical behaviours of traditional hollow sphere materials, the deformation process can generally be divided into a linear elastic stage, a large deformation stage (or plateau stage), and a densification stage. As shown in Figure 4, the elastic stage is relatively short. When the deformation reaches the initial yield stage, for different PHSSs, the buckling of weak sphere walls in one layer leads to the first local collapse deformation, and the stress decreases (① in Figure 4, Figure 5, Figure 6 and Figure 7). As the displacement increases, hollow spheres contact each other in the layer where buckling develops; as a result, the stress increases, and energy accumulates for the next collapse deformation. This deformation mechanism causes the SC and BCC PHSSs to undergo a deformation process in the initial stage of large deformation, which features local, layer-by-layer continuous buckling. Note that plastic rings form in SC structures during this stage. When a preliminary compaction state (② in Figure 4, Figure 5 and Figure 6) is achieved in which all vertically adjacent hollow spheres are in contact with each other, under continuous uniaxial loading, the hollow spheres inside the structure are interactively compressed layer by layer, and the stress increases slowly (③ in Figure 4, Figure 5 and Figure 6). For the FCC PHSS, the large deformation stage features local, layer-by-layer continuous collapse and compaction, and in this stage, the stress fluctuates smoothly (①②③ in Figure 4 and Figure 7). Finally, the different PHSSs are substantially squashed, and the space inside the structure is further squeezed; as a result, the sphere-wall contacts, especially the internal sphere-wall contacts, expand, and the material enters the densification stage, in which the stress begins to increase rapidly (④ in Figure 4, Figure 5, Figure 6 and Figure 7). After the compression test, the PHSSs were unloaded and left standing for half an hour. The BCC and FCC PHSSs regained 60–70% of their original height, while the SC PHSS was substantially crushed and failed to rebound, as shown in Figure 8.

In this study, compression tests were conducted for three groups of structures with different arrangements, and the results of each group reveal favourable consistency. A comparison between the experimental results and the numerical simulation results shows certain differences between the two, mainly because some errors and defects inevitably occurred during 3D printing and post-processing, thereby resulting in changes in the mechanical properties of the structure. The presence of errors and defects is also a common problem to be solved in the engineering application of 3D printing technologies. Overall, the deformation patterns of the PHSSs in different compression stages and the corresponding stress–strain curves were substantially consistent; therefore, the FEM established in this study is reliable.

### 3.2. Effect of Geometric Parameters

According to the results mentioned above, all large deformation behaviours of the PHSSs under quasi-static uniaxial compression began locally. To facilitate discussion and comparison, the first peak stress *Y*_P_ is defined as the first local maximum on the nominal stress–strain curve and serves as one of the indicators for predicting the large deformation behaviour; the mean stress in the plateau stage (also known as “plateau stress”) in the nominal stress (*σ*)–strain (ε) curve is also an important indicator for evaluating the large deformation behaviour of porous materials or structures; in addition, the SEA, i.e., the energy absorption per unit of mass, is one of the most important indicators of the energy absorption capacity for lightweight engineering applications. The plateau stress $\sigma_{pl}$ and SEA are defined as Equations (2) and (3), respectively.

(2) $$\sigma_{pl}=\frac{\int_{\varepsilon_{Y}}^{\varepsilon_{D}}\sigma \left(\varepsilon \right)d\varepsilon }{\varepsilon_{D}-\varepsilon_{Y}}$$

(3) $$\mathrm{SEA}=W_{m}=\frac{\int Fd\delta }{m}=\frac{\int_{0}^{\varepsilon }\sigma \left(\varepsilon \right)d\varepsilon }{\rho }$$

where $\varepsilon_{Y}$ and $\varepsilon_{D}$ generally correspond to the strains at the yield stage and the densification stage, respectively. *F* represents the reacting force of the loading plate; *δ* denotes the displacement of the loading end; *m* is the mass of the porous material; and *ρ* is the density. In this study, $\varepsilon_{D}$ is determined as the strain corresponding to the stationary point in the efficiency-strain curve where the efficiency *E*(ε) provides the last local maximum [29]:

(4) $$\frac{dE\left(\varepsilon \right)}{d\varepsilon }{|}_{\varepsilon =\varepsilon_{D}}=\frac{d\left(\int_{0}^{\varepsilon }\sigma \left(\varepsilon \right)d\varepsilon /\sigma \right)}{d\varepsilon }{|}_{\varepsilon =\varepsilon_{D}}=0$$

In the calculation of SEA, because the densification strain varies with geometric parameters, the strain ε in Equation (3) was set as 0.7 for comparison.

Based on finite element simulations, the effect of geometric parameters, such as wall thickness, hole diameter, and arrangement pattern, on the mechanical response, deformation mode, and energy absorption properties of PHSSs under quasi-static compression was investigated. Because of the localization of large deformations in hollow sphere structures, the representative volume element (RVE) under periodical or symmetric boundary conditions [4,7,11,12,15] is unsuitable for the study of the whole large deformation process. Thus, simulation calculations were performed using the multi-cell structure numerical model with five cells at each dimension according to a convergence study on the cell number. All FEMs were established by using a similar method, described in Section 2.

#### 3.2.1. Wall Thickness

**Figure 9:**
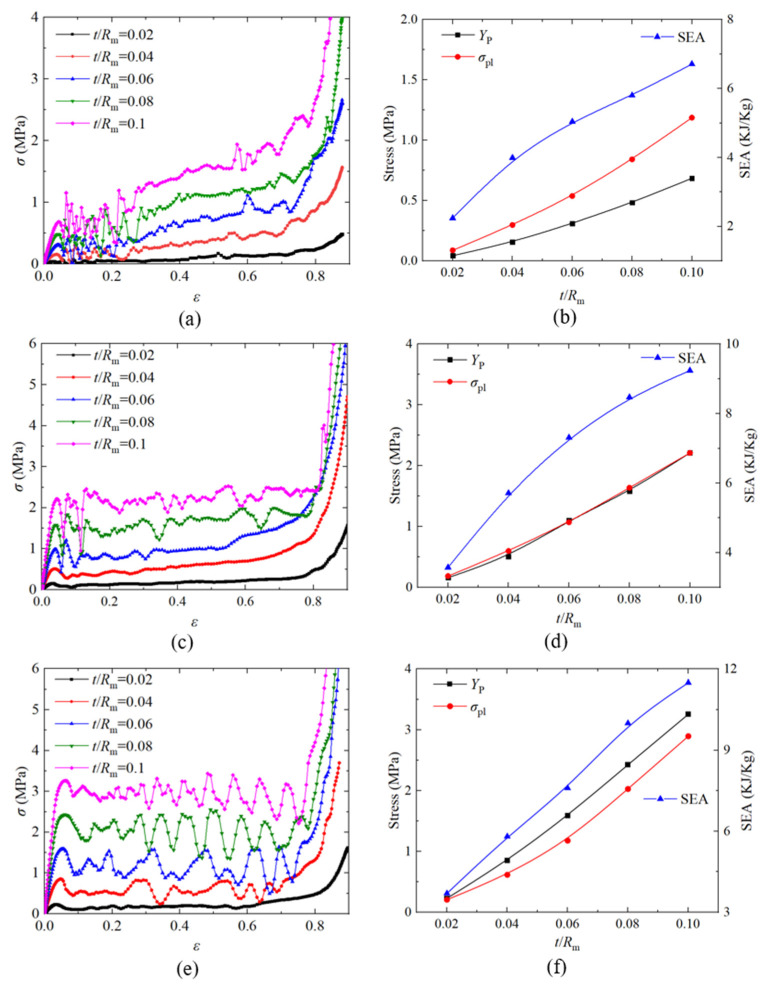
Effect of wall thickness on the nominal stress -strain curve and first peak stress *Y*_P_, plateau stress $\sigma_{pl}$ and SEA of the (**a**,**b**) SC; (**c**,**d**) BCC and (**e**,**f**) FCC structures.

When investigating the influence of wall thickness *t*, the *t* value was set as 0.195 mm, 0.39 mm, 0.585 mm, 0.78 mm, and 0.975 mm; the *d_m_* value was fixed to 5.5 mm; other parameters were unchanged; and the corresponding *t/R*_m_ was 0.02, 0.04, 0.06, 0.08, and 0.1. Figure 9a,c,e shows the nominal stress–strain curves of the SC, BCC, and FCC PHSSs, respectively, with different wall thicknesses under quasi-static compression; Figure 9b,d,f shows the curves describing the variations in first peak stress *Y*_P_, plateau stress *σ*_pl_, and SEA with different wall thicknesses for the SC, BCC, and FCC PHSSs, respectively. The results indicate that the *Y*_P_, *σ*_pl_ and SEA values of the different PHSSs increased as the wall thickness increased, and the differences were as follows: for the SC PHSS, *Y*_P_ was always less than *σ*_pl_; for the BCC PHSS, *Y*_P_ was essentially equal to *σ*_pl_; and for the FCC PHSS, *Y*_P_ was always greater than *σ*_pl_. 

**Figure 10:**
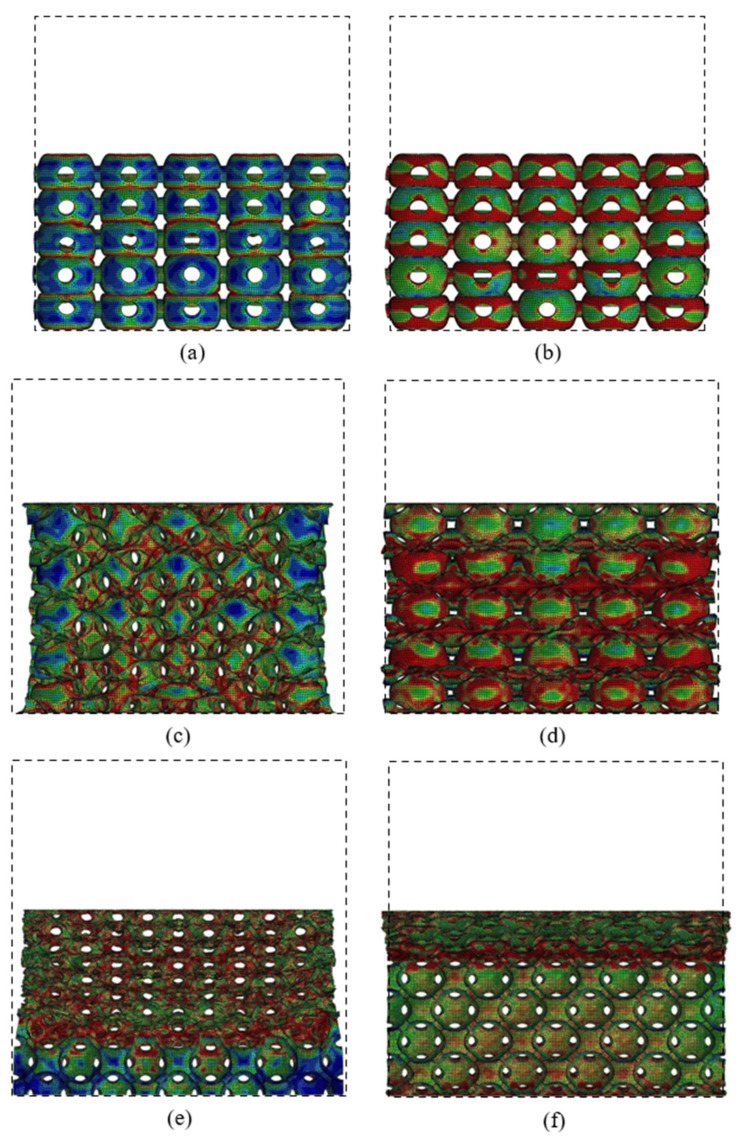
Deformations of SC structure with the (**a**) smallest and (**b**) largest wall thicknesses at a strain of 43%; deformations of BCC structure with the (**c**) smallest and (**d**) largest wall thicknesses at a strain of 37%; deformations of FCC structure with the (**e**) smallest and (**f**) largest wall thicknesses at a strain of 44%.

Figure 10 shows the large deformation patterns of the PHSSs in different arrangements, and the dotted box in the figures represents the original structural dimensions. The numerical simulation results also indicate that when the wall was very thin (the *t/R*_m_ values were 0.02 and 0.04), the sphere wall near each opening in the PHSS was prone to stress concentration; as the displacement increased, for two adjacent hollow spheres, one could undergo unstable buckling deformation, and the other could collapse into it; and for the different PHSSs, such a local deformation mechanism could cause different changes in the lateral dimensions of the structure. For the SC PHSS, the hollow spheres collapsed into each other in the same direction as the loading, and the hollow spheres squeezed each other vertically, which could increase the lateral size. When the wall was very thin, the lateral dimension did not change significantly; as the *t* value increased (*t/R*_m_ reached 0.06), the deformation mechanism of the mutual depression of the SC PHSS became stable, and the lateral dimension after compression gradually increased as *t* increased (Figure 10a,b). For the BCC and FCC PHSSs, when the wall thickness *t* was very small, under axial compression, the local buckling/depression due to the mutual extrusion of the hollow spheres caused the transverse contraction of the entire structure (*t/R*_m_ was 0.02; Figure 10c,e); as *t* gradually increased, the stress concentration and local buckling/depression of the sphere wall around the opening decreased; and when the wall was thick, the layer-by-layer bending deformation of the spherical wall dominated, while the transverse contraction of the overall structure gradually disappeared (*t/R*_m_ was 0.1; Figure 10d,f).

On the whole, for the different PHSSs, when the hollow sphere wall was extremely thin (*t/R*_m_ was 0.02 and 0.04), layer-by-layer deformation developed first to reach a preliminary compaction state (i.e., the adjacent hollow spheres were in contact with each other), and then the hollow spheres entered another stage of large deformation featuring layer-by-layer compaction (i.e., the hollow spheres squeezed each other) under uniaxial compression. As the *t* value increased (*t/R*_m_ was 0.06, 0.08, and 0.1), such deformation mode for the SC and BCC PHSSs tended to be stable, while the large deformation of the FCC PHSS was characterised by layer-by-layer continuous collapse and compaction (Figure 10f). Finally, the overall structure was further compacted under continuous uniaxial loading (i.e., the densification stage), and the stress increased sharply (Figure 9a,c,e).

**Figure 11:**
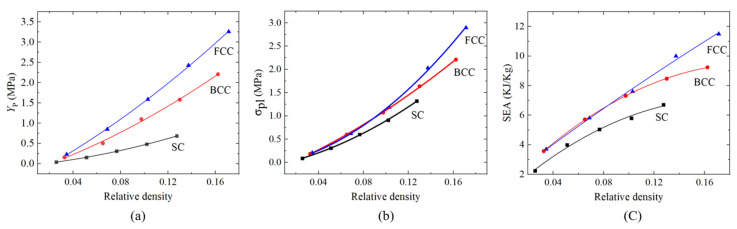
Effect of the relative density on the (**a**) first peak stress *Y*_P_, (**b**) plateau stress *σ*_pl_ and (**c**) SEA for PHSSs in different arrangements.

For the comparison of the compressive mechanical properties and energy absorption characteristics of the PHSSs in different arrangements, the numerical simulation results were fitted as a function of the relative density, as shown in Figure 11. The results show that the first peak stress *Y*_P_ and plateau stress *σ*_pl_ of the PHSSs increased as the relative density increased; the lower the relative density was, the closer the results of the different PHSSs were to one another. Overall, with the same relative density, the *Y*_P_, *σ*_pl_, and SEA values of the SC PHSS were the lowest, the *Y*_P_ value of the FCC PHSS was the highest, and the values of the BCC PHSS were in between. The possible causes are as follows: the individual hollow sphere inside the SC PHSS was subject to the least constraints; the individual hollow sphere inside the FCC PHSS was under the most constraints; and the constraints of the BCC PHSS were in between. The more constraints a hollow sphere is under, the harder it is to buckle. When the relative density was between 0.02 and 0.1, the *σ*_pl_ and SEA values of the BCC PHSS were extremely close to those of the FCC PHSS. When the relative density exceeded 0.1, the *σ*_pl_ and SEA values of the FCC PHSS progressively exceeded those of the BCC PHSS.

#### 3.2.2. Hole Diameter

**Figure 12:**
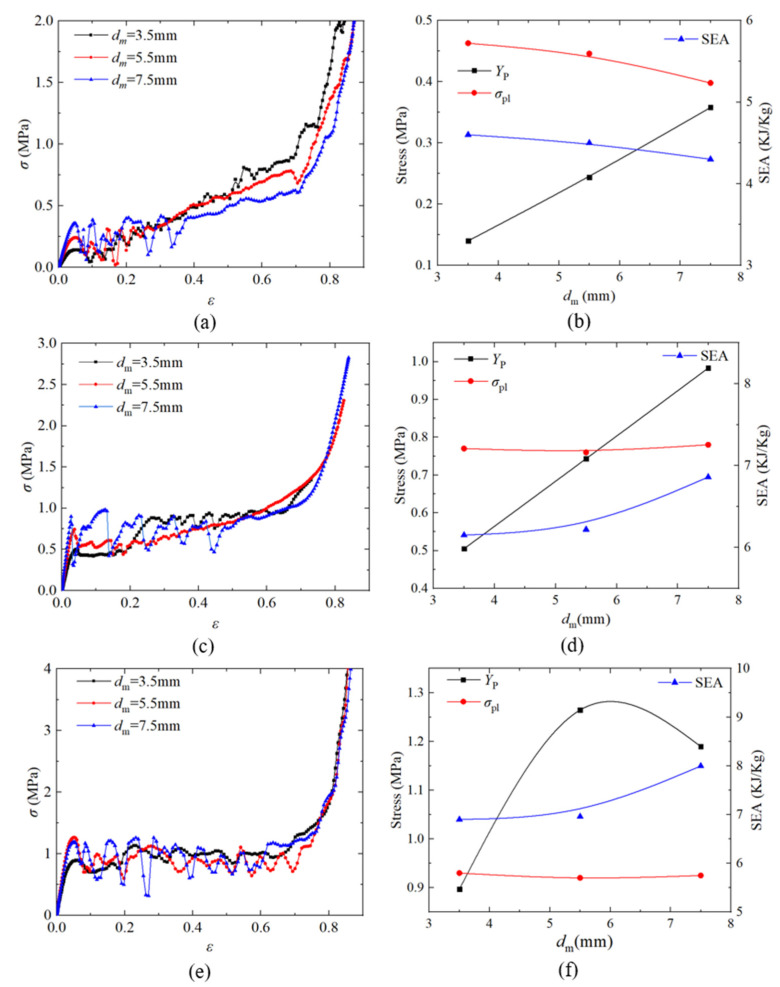
Effect of hole diameter on the nominal stress–strain curve and first peak stress *Y*_P_, plateau stress $\sigma_{\mathrm{pl}}$ and SEA of (**a**,**b**) SC; (**c**,**d**) BCC and (**e**,**f**) FCC structures.

When investigating the effect of hole diameter *d_m_*, the *d_m_* value was 3.5 mm, 5.5 mm, and 7.5 mm, the *t* value was fixed to 0.5 mm, and the other parameters were unchanged. Figure 12a,c,e shows the nominal stress–strain curves of the SC, BCC, and FCC PHSSs, respectively, with different hole diameters under quasi-static compression; Figure 12b,d,f show the curves describing the variations in first peak stress *Y*_P_, plateau stress *σ*_pl_ and SEA with the change in the hole diameter for the SC, BCC, and FCC PHSSs, respectively. The results show that for the SC and BCC PHSSs, the *Y*_P_ value increased as *d_m_* increased; i.e., *Y*_P_ was clearly positively correlated with *d_m_*, while for the FCC PHSS, when *d_m_* increased to a certain value (approximately 6 mm by fitting), *Y*_P_ began to decrease, and the curve reflecting the variation in *Y*_P_ of the FCC PHSS with the change in *d_m_* was parabolic, which is probably because the FCC PHSS had the most openings; therefore, the larger *d_m_* was, the greater the loss of mass was, and when *d_m_* reached approximately 6 mm, the strength began to decrease due to an excessively high mass loss. Similar conclusions have been drawn for the mechanical properties in the elastic stage and the initial yield stage of PHSSs [27]. In addition, the hole diameter had a significant effect on the *Y*_P_ value of PHSSs in different arrangements, but it had a relatively small effect on the *σ*_pl_ and SEA values in the large deformation stage of various PHSSs (due to the average effect in the stress–strain curves). The SEA results show that for the SC PHSS, the SEA value decreased slightly as *d_m_* increased, but the energy absorption did not fluctuate significantly. For the BCC and FCC PHSSs, when *d_m_* = 7.5 mm, the SEA value was significantly higher than the values of structures with smaller hole diameters (3.5 mm, 5.5 mm). This may be because the BCC and FCC PHSSs had more openings than the SC PHSS; when the *d_m_* value was very large, the decrease in the density was greater, and according to Equation (3), the SEA value of the BCC and FCC PHSSs could be higher for large *d_m_* values. 

**Figure 13:**
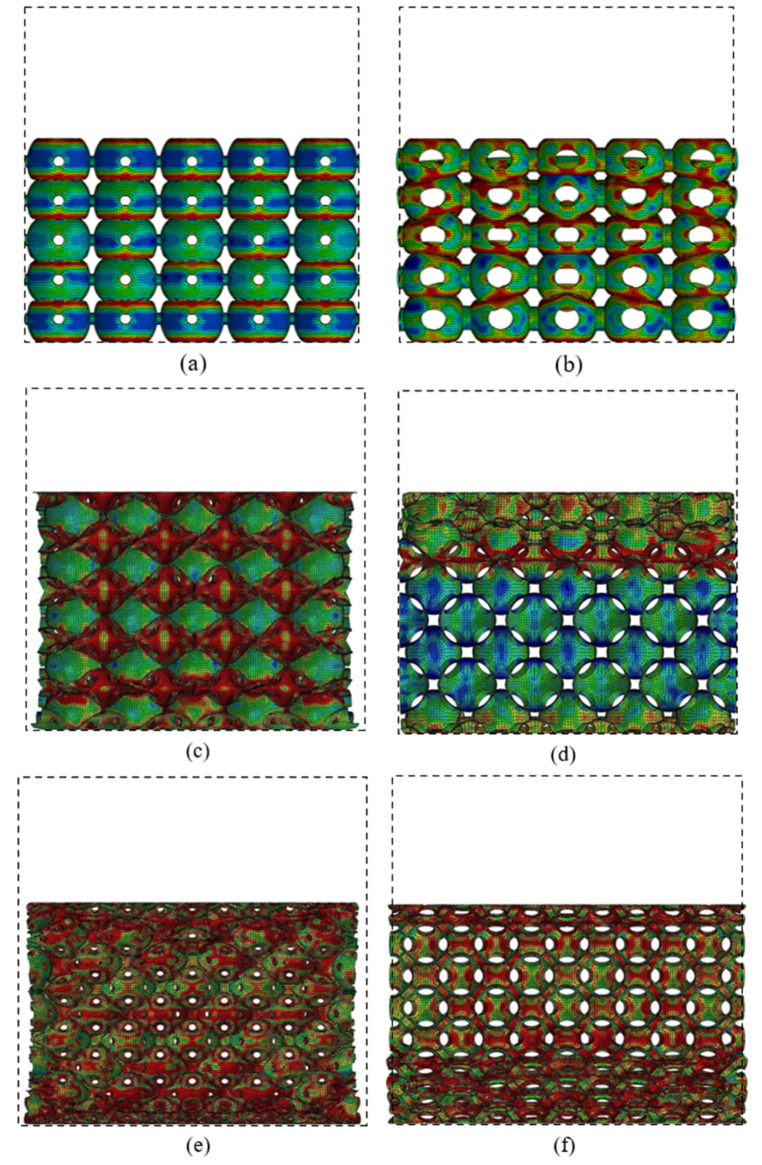
Deformations of SC structure with the (**a**) smallest and (**b**) largest hole diameters at a strain of 38%; deformations of BCC structure with the (**c**) smallest and (**d**) largest hole diameters at a strain of 28%; deformations of FCC structure with the (**e**) smallest and (**f**) largest hole diameters at a strain of 36%.

Figure 13 shows the deformation modes of PHSSs in different packing patterns with the smallest (*d_m_* = 3.5 mm) and largest (*d_m_* = 7.5 mm) hole diameters. The influence mechanism of hole diameter *d_m_* was similar to that of wall thickness t, i.e., the sphere wall near the opening in the different PHSSs was prone to stress concentration and local buckling/depression when *d_m_* was very small; the minor changes in the lateral dimensions of the SC PHSS could almost be ignored (zero expansion) (*d_m_* was 3.5 mm; Figure 13a); and obvious transverse contraction developed in the BCC and FCC PHSSs (*d_m_* was 3.5 mm; Figure 13c,e). As *d_m_* increased, the stress concentration and local buckling or inward depression of spheres in BCC and FCC structures were significantly improved, and the major deformation was the layer-by-layer bending behaviour of the sphere walls; moreover, the transverse contraction of the entire structure gradually disappeared (*d_m_* was 7.5 mm; Figure 13d,f).

### 3.3. Comparison with the Traditional MHSS

**Figure 14:**
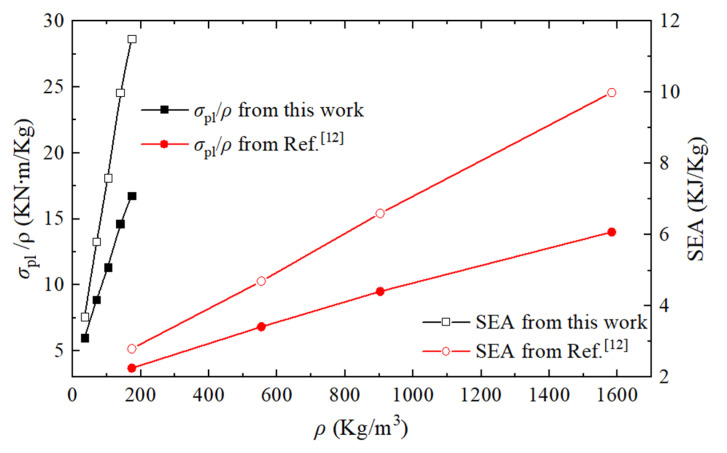
Comparison of *σ*_pl_/*ρ* and SEA of the PHSS in this work with the MHSS in Reference [12].

The specific strength (plateau stress/density, *σ*_pl_/*ρ*) and energy absorption characteristics (SEA) of PHSS are compared with those of the traditional MHSS made of mild steel, as shown in Figure 14. The material is defined to be elastoplastic with a Young’s modulus of 200 GPa and a yield stress of 200 MPa. The density *ρ* of the porous structure or material is the product of the relative density and solid material density. This comparison focuses on the results from FCC structures with different wall thicknesses according to Reference [12]. The presented 3D-printed PHSS has a lower density ranging from 34–174 kg/m^3^, and the traditional MHSS has a higher density ranging from 173 to 1600 kg/m^3^, while the specific strength and SEA capacities of the 3D-printed PHSS are higher than those of the traditional MHSS. The reason can be attributed to two aspects: using lightweight solid material with good mechanical properties and perforating appropriately in hollow sphere structures for further dead weight reduction.

## 4. Conclusions

For this study, PHSSs in three different arrangements were fabricated using MJF 3D printing technology, and the mechanical behaviours of these PHSSs under quasi-static compression were studied through experiments and finite element simulations. The experimental results verify the effectiveness of the FEM. The results show that the deformation process of the 3D-printed PHSSs was similar to that of traditional hollow sphere structures and consisted of three stages, i.e., an elastic deformation stage, a large deformation stage, and a densification stage. Special attention was given to the large deformation stage, which is directly related to the energy absorbing capacity. Based on the numerical simulation, the effects of the geometric parameters of the PHSSs, such as the sphere arrangement, wall thickness, and hole diameter, on the compressive mechanical behaviours and energy absorption characteristics were investigated. The following conclusions can be drawn:

(1) With different geometric parameters, the SC and BCC PHSSs underwent a preliminary compaction sub-stage after layer-by-layer buckling in the stage of large deformation; the FCC PHSS did not exhibit a similar deformation behaviour unless the *t* or *d_m_* value was extremely small; and the layer-by-layer collapse-compaction behaviour was dominant for the large deformation of the FCC PHSS.
(2) Among the PHSSs with the same relative density, the FCC PHSS exhibited the best compressive mechanical properties and energy absorption capacity; the SC PHSS exhibited the lowest; and the BCC PHSS was in between.
(3) The first peak stress, plateau stress and SEA of the PHSSs increased as the wall thickness t increased, and the hole diameter *d_m_* had a significant effect on the first peak stress of different PHSSs but a relatively small effect on the plateau stress and SEA.
(4) When the wall thickness was very thin or the hole diameter was very small, the local depression of hollow spheres caused the BCC and FCC PHSSs to exhibit obvious transverse contraction under axial compression, and this behaviour weakened as the wall thickness or hole diameter increased.
(5) The presented 3D-printed PHSS was much lighter than the traditional MHSS made of mild steel and had higher specific strength and SEA than the traditional MHSS.

## Figures and Tables

**Table 1:** Geometrical parameters and relative densities.

Geometrical Parameter		Relative Density *ρ_rl_*		
*t*/mm	*d_m_*/mm	SC	BCC	FCC
0.195	5.5	0.02553	0.03253	0.0344
0.39		0.05105	0.06504	0.06875
0.585		0.07654	0.0975	0.10303
0.78		0.10207	0.13	0.13731
0.975		0.12746	0.16229	0.17129
0.5	3.5	0.06756	0.08706	0.09434
	5.5	0.0654	0.08332	0.08822
	7.5	0.06274	0.07874	0.0807

## Data Availability

Not Applicable.
